# Perspectives of patients with depression and chronic pain about bone health after a fragility fracture: A qualitative study

**DOI:** 10.1111/hex.13361

**Published:** 2021-09-27

**Authors:** Joanna E. M. Sale, Monique Gignac, Lucy Frankel, Stephen Thielke, Earl Bogoch, Victoria Elliot‐Gibson, Gillian Hawker, Larry Funnell

**Affiliations:** ^1^ Musculoskeletal Health and Outcomes Research, Li Ka Shing Knowledge Institute, St. Michael's Hospital Unity Health Toronto Toronto Ontario Canada; ^2^ Institute of Health Policy, Management and Evaluation University of Toronto Toronto Ontario Canada; ^3^ Department of Surgery, Faculty of Medicine University of Toronto Toronto Ontario Canada; ^4^ Institute for Work & Health Toronto Ontario Canada; ^5^ Psychiatry and Behavioral Sciences University of Washington Seattle Washington USA; ^6^ Department of Surgery, St. Michael's Hospital, Unity Health Toronto University of Toronto Toronto Ontario Canada; ^7^ Brookfield Chair Toronto Ontario Canada; ^8^ Faculty of Medicine University of Toronto Toronto Ontario Canada; ^9^ Osteoporosis Canada Toronto Ontario Canada

**Keywords:** bone health, chronic pain, depression, fragility fracture, patient perspective

## Abstract

**Background:**

Compromised bone health is often associated with depression and chronic pain.

**Objective:**

To examine: (1) the experience of existing depression and chronic nonfracture pain in patients with a fragility fracture; and (2) the effects of the fracture on depression and pain.

**Design:**

A phenomenological study guided by Giorgi's analytical procedures.

**Setting and Participants:**

Fracture patients who reported taking prescription medication for one or more comorbidities, excluding compromised bone health.

**Main Variables Studied:**

Patients were interviewed within 6 weeks of their fracture, and 1 year later. Interview questions addressed the recent fracture and patients' experience with bone health and their other health conditions, such as depression and chronic pain, including the medications taken for these conditions.

**Results:**

Twenty‐six patients (5 men, 21 women) aged 45–84 years old with hip (*n* = 5) and nonhip (*n* = 21) fractures were recruited. Twenty‐one participants reported depression and/or chronic nonfracture pain, of which seven reported having both depression and chronic pain. Two themes were consistent, based on our analysis: (1) depression and chronic pain overshadowed attention to bone health; and (2) the fracture exacerbated reported experiences of existing depression and chronic pain.

**Conclusion:**

Experiences with depression and pain take priority over bone health and may worsen as a result of the fracture. Health care providers treating fragility fractures might ask patients about depression and pain and take appropriate steps to address patients' more general emotional and physical state.

**Patient Contribution:**

A patient representative was involved in the study conception, data interpretation and manuscript writing.

## 1 INTRODUCTION

Compromised bone health is often associated with depression and chronic pain. There is evidence to suggest a bidirectional relationship between bone health and depression. Specifically, researchers have suggested deleterious effects of depression on bone health through bone loss and increased fracture risk.^1^ The causal mechanism is unclear but possibly due to depression altering concentrations of many hormones that affect bone formation and/or resorption, such as cortisol.^1^ Medications taken for depression may also be detrimental to bone.^2^, ^3^, ^4^ For example, serotonin can influence bone metabolism as serotonin receptors and transporters are present in osteoblasts and osteoclasts.^4^ At the same time, fragility fractures often precede the onset of depression.^5^, ^6^ In one cohort study, 10% of individuals reported depressive symptoms after a hip fracture.^6^

There is also evidence of a bidirectional relationship between bone health and chronic nonfracture pain. Medications taken for chronic pain, such as nonsteroidal antiinflammatory drugs (NSAIDs) and opioids, including codeine, can have negative bone health effects, including increased fracture risk, increased risk of falling and decreased bone mineral density.^2^, ^7^, ^8^ The mechanism underlying this effect is unclear.^7^, ^8^ Fragility fractures, such as vertebral fractures, can also result in long‐term pain at the site of the fracture,^9^, ^10^ which is thought to be due to physiological changes in the spine and loss of height.^11^, ^12^ Other types of fractures have also been shown to result in reports of long‐term pain at the site of the fracture,^13^, ^14^ possibly due to the development of arthritis at the fracture site.^15^, ^16^ Little is known about whether fragility fractures have an effect on chronic pain conditions unrelated to the fracture.

Few researchers have examined the co‐occurrence of depression and chronic nonfracture pain in patients who have had a fragility fracture, and what factors may worsen these conditions. In one randomized controlled trial of patients with a wrist fracture, the most common reported comorbidities included osteoarthritis (34%–42%) and depression (11%–15%).^17^, ^18^ Although not determined in that study, it is expected that reports of chronic joint pain would have been common in most of the patients reporting osteoarthritis. Kelly‐Pettersson and colleagues reported that approximately 22% of hip fracture patients reported depressive symptoms at baseline,^19^ but it is unclear whether the depressive symptoms existed at the time of hip fracture or occurred after the hip fracture was sustained.

Our purpose was to examine experiences of depression and chronic nonfracture pain in patients who presented with a fragility fracture and other comorbidities. A fragility fracture is one that occurs after a slip, trip or fall from standing height or less.^20^ This is distinct from high‐trauma fractures, which typically result from motor vehicle crashes and falls from greater than standing height.^21^ Fragility fractures are a sign of poor bone quality and a predictor of future fractures.^22^, ^23^ A Fracture Liaison Service is a model of care that identifies individuals over the age of 50 years who present to a hospital with a fragility fracture to ensure they receive a fracture risk assessment and bone health treatment according to current clinical practice guidelines.^24^ Treatment includes pharmacological and nonpharmacological treatment, such as vitamin D supplementation, adequate calcium intake, and exercise.^25^ Pharmacological treatment has been shown to reduce refracture^26^, ^27^, ^28^ and mortality rates.^28^ We anticipated that our investigation might partially contribute to our understanding of the modest uptake of bone health recommendations after a fragility fracture, such as taking bone active medication.^29^, ^30^ In one systematic review, less than 35% of individuals initiated medication after a postfracture intervention.^31^ Depression has been shown to be associated with poor adherence to bone active medication.^32^

Using a qualitative approach and relying on patients' perspectives, we sought to understand the complexity and meaning of bone health in comparison to that of chronic pain and depression. Previous qualitative studies in bone health have demonstrated that patients have limited knowledge of bone densitometry and bone health^33^ and are unclear about testing and treatment recommendations.^34^, ^35^ Studies have shown that the circle of care for those with fragility fractures is disrupted at vital communication junctures^36^ and that patients perceive inconsistent messages within, and across, primary care providers and bone specialists.^37^ One qualitative synthesis demonstrated that individuals create meaning of an osteoporosis diagnosis based on self‐perceived risk, self‐perceived severity of osteoporosis and self‐perceived health.^38^ Authors have also reported that patients do not connect their fractures to bone health,^39^, ^40^ that many patients classified as ‘high risk for future fracture’ do not believe they are high risk,^41^ and that having caregiving responsibilities affect the management of fragility fractures.^42^ Specifically, our objectives were to examine: (1) the experience of depression and chronic nonfracture pain in patients with a fragility fracture; and (2) the effects of the fracture on depression and chronic pain.

## 2 METHODS

This study was part of a larger study examining bone health management in patients with a fragility fracture who reported one or more comorbidities. It involved a 2‐year qualitative investigation guided by phenomenology as conceptualized by Giorgi and Wertz (the ‘Duquesne school’).^43^, ^44^, ^45^ Phenomenology was relevant to our study design as it emphasizes the importance of direct experiences, perceptions and actions.^46^, ^47^ Ethical approval for the study was received by Unity Health Toronto (REB#: 14‐301).

We recruited patients from a Fracture Liaison Service serving approximately 430 patients annually in a Canadian urban hospital.^48^, ^49^ Consistent with phenomenology, we employed criterion sampling^50^ where eligible individuals were English‐speaking men and women, 45+ years old, who self‐reported currently taking prescription medication for at least one additional chronic health condition (bone health could not be the only chronic condition for which patients were taking medication). To aid with recruitment, we created a list of eligible chronic health conditions, such as arthritis and high blood pressure, from two Canadian sources^51^, ^52^ and expanded it to include other conditions such as those causing secondary bone loss. Participants were not eligible if they only reported conditions on the list of excluded conditions (see Table 1). Patients who exhibited cognitive difficulties that might compromise their ability to give informed consent or participate in an interview were not approached.

**Table 1:** Conditions determining eligibility criteria

Conditions included
Anxiety disorder/mood disorder
Arthritis
Asthma/chronic obstructive pulmonary disease
Back problems (excluding arthritis)
Bowel disorder (irritable bowel syndrome, Crohn's disease, ulcerative colitis)
Cancer
Cardiovascular disease
Cataracts
Chronic bronchitis
Chronic fatigue syndrome
Depression
Diabetes
Dizziness
Endocrine conditions
Epilepsy
Fibromyalgia
Food allergies
Gastrointestinal conditions
Glaucoma
High blood pressure
High cholesterol
Human immunodeficiency virus
Hypogonadism
Impaired depth perception
Impaired mobility
Migraine headaches
Mood disorder
Multiple chemical sensitivities
Premature ovarian failure
Renal impairment
Stomach/intestinal ulcers
Stroke (effects of)
Thyroid condition
Urinary incontinence
Conditions excluded
Attention deficit disorder
Haematuria
Herpes
Hormone imbalance
Kidney transplant
Lung infection
Severe anaemia (needing blood transfusion)
Sleep apnoea/insomnia

A fracture prevention coordinator (V. E.‐G.) identified potentially eligible participants and determined their interest in participating in our study. The coordinator screens patients with fragility fractures and facilitates treatment with bone active medication according to the Canadian Clinical Practice Guidelines for the Diagnosis and Management of Osteoporosis.^25^ A female study coordinator (L. F.) with 13 years of qualitative research experience and a Bachelor of Arts degree telephoned patients to obtain research consent, confirm eligibility based on patients' self‐report of existing chronic health conditions, and schedule baseline interviews (60–90 min). Follow‐up interviews (approximately 30 min) were scheduled approximately 1 year after the baseline interview. We conducted the majority of baseline interviews in patients' homes within 6 weeks of their fracture and follow‐up interviews by telephone. Topic areas in the baseline interview guide addressed the recent fracture and patients' experiences with bone health and their other health conditions, such as depression and conditions that might be associated with chronic pain, including the medications taken for these conditions. For example, we asked patients about the condition(s) of concern to them at the moment and about the importance of bone health in comparison with their other health conditions. We also asked how bone health recommendations might affect the management of these other conditions. The second interview focused on changes, if any, to patients' experiences with bone health and their other health conditions as well as any changes in medication regimens (see Table 2).

**Table 2:** Interview guides

*First interview*		
1	Tell me about your fracture.	
	What changes, if any, has your fracture had on your overall health?	
	For example, do you have any ongoing pain as a result?	
2	What were you told about your bone health after your fracture?	
	What do you understand about your bone health?	
	Did you have any tests (e.g., a BMD test) for bone health? Describe. What did these tests tell you?	
	What did the fracture clinic/your family doctor/your specialist say?	
	What recommendations did you receive from the fracture clinic/your family doctor/your specialist?	
	How does your bone health affect your life (e.g., mobility, personal care, participation in social and recreational activities)?	
	Is your bone health a serious issue for you? Why/why not?	
	How does your bone health condition make you feel?	
3	We would like to know about your overall health. What other conditions are you being treated for? Tell me about them (*if the participant has more than one condition, go through each condition*)	
	Do you currently have any symptoms for [condition(s) mentioned]? Explain.	
	Who have you seen for [condition(s) mentioned]?	
	What has your family doctor or specialist told you about [condition(s) mentioned]?	
	What motivates you to take care of [condition(s) mentioned]?	
	What kinds of tests have you had for [condition(s) mentioned]?	
	What did these tests tell you?	
	Have you ever been hospitalized for [condition(s) mentioned]? Explain.	
	Is [condition(s) mentioned] a serious issue for you? Why/why not?	
	How do/does [condition(s) mentioned] affect your life, if at all (e.g., mobility, personal care, participation in social and recreational activities)?	
	How do these conditions make you feel?	
4	How does [condition mentioned] affect your bone health? (*if the participant has more than one condition, go through each condition*)	
	Have your other health conditions impacted your ability to implement treatment for bone health? Explain.	
	Is there anything about [condition(s) mentioned] that affects your bone health/makes it easy or difficult to implement recommendations for your bone health? For example, people with high cholesterol have told us that their calcium recommendations for their bone health conflict with the low‐fat diet recommendations that they have been given for lowering their cholesterol levels	
	Do any of your other conditions influence in any way how you have been approaching your bone health?	
5	I asked you how your [condition named] affects your bone health. What about the opposite, how does your bone health affect your [condition named]? (*if the participant has more than one condition, go through each condition*)	
6	We are trying to understand how people deal with having several health conditions at the same time. Do you ever feel like you have to choose between your bone health and another condition that you have? Explain	
	How important is bone health in comparison to your other health conditions and why?	
	How do you feel about the treatment recommendations for bone health compared with treatment for your other condition(s)?	
	How do you feel about [medication/supplements] for bone health compared with those recommended for your other condition(s)?	
	Do the symptoms of any one condition affect how you feel and what you do about that condition/those conditions?	
7	Tell me about your daily routine for managing your [condition(s) mentioned]?	
	Tell me about any supplements/medications that you are taking?	
	What else are you doing to look after [condition(s) mentioned], for example, exercises?	
	How often do you forget to follow recommendations for your [condition(s) mentioned]?	
	What makes it easy/difficult for you to follow these recommendations for [condition(s) mentioned]?	
	Do your other medications/conditions affect your ability to include bone health in your daily routine? Why/why not?	
	What would make bone health more or less serious in relation to your [condition(s) mentioned]?	
*Second interview*		
1	Tell me about your fracture since I last interviewed you.	
	How is your fracture now?	
	Have you had any new fractures? If so, tell me about it/them?	
	Who have you seen about your bone health since our interview a year ago? Tell me about those experiences.	
	Have there been any changes to your bone health in the last year? (since I saw you on [date])? Explain.	
2	<If there have been changes in bone health/bone health recommendations/actions regarding bone health>Tell me a little about the changes in your bone health? (*repeat examples provided by the participant*)	
	How have these changes impacted your general health?	
	Do you anticipate that these changes will impact your general health?	
	Have your other health conditions impacted your ability to implement these new changes or recommendations? Explain.	
	What has made it difficult for you to make these changes?	
3	<If no changes in bone health/bone health recommendations/actions regarding bone health>Tell me about what is going on with your bone health these days?	
	What is your understanding of it?	
	What are you doing about your bone health?	
4	Have there been any changes in your [other condition(s) mentioned in the previous interview] since [date of the last interview]?	
	Tell me about it/them.	
	Have you had any new tests related to this/these conditions? If so, what did the test results say?	
	What are you currently doing about this/these conditions?	
	Have you been hospitalized for any of these conditions? Please explain.	
	Do you feel differently about your [condition(s)] this year compared to last year? Explain.	
	Has anything happened in your life that makes you think differently about any of your health conditions?	
	Do you feel differently about your bone health in light of changes to [condition(s) mentioned]? For example, have you had to make any changes in how you look after your bone health because of changes in [condition(s) mentioned]?	
5	Have you been diagnosed or treated for any *new* health conditions in the last year (since I interviewed you on [date])?	
	Tell me about it/them?	
	Have you had any tests related to these conditions? If so, what did the test results say?	
	What has your family doctor or specialist told you about this condition(s)?	
	What are you doing for this/these new health condition(s)? For example, are you taking any new medications?	
	How easy or difficult has it been to incorporate this new condition, and treatment for it, into your daily life?	
	Has this condition(s) caused you to make any general changes in your life?	
	How do you feel about your bone health in light of your new condition(s)?	
	How important is [all current and new conditions mentioned] compared to your bone health? Why?	

In phenomenological studies, the outcome is a description of the essence, or structure, of what is perceived and experienced across individuals.^44^ Iterative analyses of the data began after the first two baseline interviews were conducted. Preliminary codes were identified and then revised as more interviews were conducted. Two individuals (J. E. M. S., L. F.) with qualitative expertise analysed the transcripts independently and met regularly to develop and finalize a coding template, which was then applied to all transcripts using NVivo.^53^ To promote a comprehensive examination of the data,^54^ all transcripts were coded by the two coders. The analysis was guided by Giorgi's procedures.^43^ To organize the data, meaning units, or codes, were documented in the margins, and codes relevant to our objectives were grouped together. We reflected on the relationships among the codes and developed themes that were supported by direct quotations from participants.^55^ Discussions regarding analysis and interpretation of the data were reviewed by the study team as data collection and analysis progressed. Multiple thematic possibilities were considered by the team (a critical appraisal strategy referred to as imaginative variation^44^, ^45^) and a consensus was sought on the final themes based on the relevance,^56^ novelty and clinical significance of the findings. Cases that did not fit with our general findings were discussed and reported to promote transparency in the research process.^57^

Consistent with GRIPP2‐SF,^58^ a public contributor was a formal part of the research team from the beginning of the study. He was involved in the grant‐writing process, which included the drafting of the interview guide and refining the focus of the research questions. Along with all team members, this individual was involved in discussions about the data and the findings reported (see Table 3).

**Table 3:** Patient and public involvement in the study using GRIPP2‐SF

Aim	We sought to examine: (1) the experience of depression and chronic nonfracture pain in patients with a fragility fracture; and (2) the effects of the fracture on depression and chronic pain.
Methods	We recruited one public contributor to the research team from the beginning of the study. The public contributor was involved in the grant‐writing process, which included drafting the interview guide and refining the focus of the research questions. This individual was involved in the analysis and interpretation of the deidentified data. He participated in edits to the final paper and is a coauthor.
Study results	The public contributor assessed the relevance of the findings and helped the team to develop the themes reported.
Discussion and conclusions	By participating in the study from its inception (writing the grant), the public contributor helped shape the research questions and the topic areas in the interview guide. There were limitations to the public contributor's participation. Due to research ethical concerns, this individual did not have access to the transcripts and could only comment on the deidentified data presented by the analysts.
Reflections/critical perspective	The public contributor has worked with our research team since 2010. The team's relationship with the public contributor has strengthened over the years, highlighting the importance of sustaining long‐term collaborations with public contributors. The public contributor was not trained in qualitative research, so could not comment on the methodological aspects of the study.

## 3 RESULTS

We recruited 26 patients (5 men, 21 women) aged 45–84 years old with the hip (*n* = 5), wrist (*n* = 15), spine (*n* = 1), shoulder (*n* = 1), other (*n* = 2) or multiple fractures (*n* = 2). This sample size met recommendations for phenomenological studies.^44^, ^47^ Thirteen reported a history of previous fractures (11 women, 2 men). According to the Canadian clinical practice guidelines, 16 were deemed high risk for future fracture due to previous fractures and/or a current hip or spine fracture.^25^ Four participants could not be located 1 year later for a follow‐up interview. In the majority of cases (*n* = 17), baseline interviews were conducted in patients' homes. The remaining nine interviews were conducted by telephone (*n* = 4), in the interviewer's office (*n* = 3) and at a location suggested by the participant (*n* = 2).

Participants presented with one to seven chronic health conditions in addition to compromised bone health. The most common chronic health conditions reported were depression (*n* = 12), high blood pressure (*n* = 11), arthritis (osteoarthritis and rheumatoid arthritis combined) (*n* = 10), high cholesterol (*n* = 9) and hypothyroidism (*n* = 7). Sixteen participants reported conditions resulting in chronic nonfracture pain, such as arthritis, ‘nerve pain’, sciatica and spinal stenosis (7 were currently taking prescription medication for the pain, including analgesics, opioids, NSAIDs and anticonvulsants for neuropathic pain) and 12 participants reported depression (9 were currently taking prescription medication for depression, including selective serotonin reuptake inhibitors, serotonin and norepinephrine reuptake inhibitors, norepinephrine and dopamine reuptake inhibitors, benzodiazepines, anticonvulsants and atypical antipsychotics). In total, 21 of the 26 patients reported depression and/or chronic nonfracture‐related pain, of which seven reported having both depression and chronic pain. The majority of participants with depression or chronic pain reported giving up physical and social activities due to these conditions.

Two themes were consistent, based on our analysis: (1) depression and chronic nonfracture pain overshadowed attention to bone health; and (2) the fracture exacerbated reported experiences of existing depression and chronic pain. We did not observe any differences between men and women, or in participants having different comorbidities or other characteristics, related to these two themes.

### 3.1 Depression and chronic nonfracture pain overshadowed attention to bone health

Participants reported experiencing depression and/or chronic nonfracture pain for 15 years or longer. These conditions were described as all‐consuming and they were prioritized over bone health (see Table 4).

**Table 4:** Depression and chronic nonfracture pain overshadowed attention to bone health

**ID**	**Quote**
*Depression was all‐consuming*	
4	‘I've had [depression] for years…you can't get out of the house and you can't get out with your friends…the depression really had a lot to do with the physical…it seems that as my mobility got worse…when I was younger, I had to stop riding a bike [because of juvenile rheumatoid arthritis], so slowly you stop, you can't walk as far…and it's like you're losing bits and pieces…you're like grieving each time you have a loss but it's just little bits so that's where the depression came in’ (Female, 52 years old)
5	‘the whole reason that I was referred to Dr. [X], who is a psychiatrist is because I just wanted to die…And with all these conditions…it's like, oh, why me? Is it never going to stop?’ (Female, 72 years old)
8	‘[Depression has] affected my life in the sense that, well, the way that gloom does. You don't do the things you should do. You don't stay socialized’ (Female, 84 years old)
9	‘I had decided that I'm just going to come home and kill myself. I mean, this came out of depression…I'm just tired of living alone and being alone…I have become more withdrawn here in the building, and I am aware of it’ (Male, 77 years old)
15	‘I just sometimes feel…emotional…very…high anxiety, feelings of anger inside, and then it subsides’ (Female, 63 years old)
16	‘I've been diagnosed [with depression] kind of on and off since young adulthood, but I'm aware that looking back, I think I had depressive symptoms as a child, too, and certainly as a teenager…It's a gradual process…It [depression] has a lot to do with how family treated me also and…friends that I thought were friends, how they treated you when you've got to put holes in your finger and you take injections every day…My own particular depression manifests with fatigue and inertia and kind of deep self‐contempt. You slip into this certain knowledge that you are an absolute total failure of every sort. Terrible mother, terrible scholar, useless scholar, can't even keep the house clean…So then I start to withdraw from things…I start cancelling things that I was going to do with other people. Then, I just don't go out at all and don't do anything at all’ (Female, 56 years old)
17	‘Probably [had depression] all my life, but I was formally diagnosed in 1995, maybe. Let's just say '97…and that would make it 20 years…It's hard to describe depression to people who've never experienced it because it's not sadness and it's not being blue, it's not being upset. It's a lack of hope and so the world becomes insignificant and you don't believe that anything that you do can have any impact on making your life better… sometimes you cry a lot and sometimes you have no affect, you just checked out…It [depression] comes for no apparent reason and it goes for no apparent reason that I can find…even on a good day you know that you're a hair's breadth away from not feeling good, always. You're always a little bit conscious of managing your surroundings and trying to stay cheerful…[The depression] has tainted everything, it's ruined it. I was a very successful career woman with a very big life, lots of friends< talking through tears>. Very exciting career, lots of money and now I'm just [an] aging woman with a middle wage job, hides in her apartment’ (Female, 58 years old)
18	‘I've really been depressed for a long time…I find I have no drive. I'm always sad’ (Female, 45 years old)
19	‘[I've had depression] since I was a child, for sure. I started taking medication for it in my mid‐30s…I quit [the medication], and that was a mistake, but I tried living without it for a while and I went back to all the…the horrible way I was living, never sleeping and always thinking the worst and never being able to relax…she [physician] started me on the drugs again…I used to know it was going to be a bad day if I couldn't put my left shoe on before my right shoe. It was triggers like that…[A bad day] is when I do start taking totally random things personally…I would just start to think, oh, people don't like me, because I hear that they were out and I wasn't there, that kind of thing. I start to blow things out of proportion, for sure. A really bad day, I guess, even just I overhear something on the subway and I think, “Was he talking about me?” I start projecting things that way…I am more likely to not want to go out…even if I'm asked, I'll say “no”, which is obviously counterproductive’ (Female, 61 years old)
22	‘In August 1999, I noticed I was getting depressed for, what I thought, no reason at all, to the point of crying in public’ (Male, 62 years old)
23	‘…clinical depression, eventually diagnosed…I was with that one psychiatrist for 22 years…The best way I can describe it is that I don't seem capable of feeling joy…laughter, for me, comes difficult. Tears come easier…A good day would be that I am not reduced to tears. I didn't realize this, but my older daughter, on different occasions, has seen me just sitting, looking into space. She would say, “Mom, what is wrong?” and I would say, “I don't know”, and I would start to cry…A bad day is like pouring rain…There were times that I didn't want to be with people. Getting dinner for the family, and then just ultimately getting dinner at all, I couldn't do it. It just became a burden’ (Female, 80 years old)
25	‘The depression is something that I guess it's always been there. [My depression is] a cloud…[under the cloud] I don't do anything. I have to have almost no food or cigarettes in the house to force me out’ (Male, 57 years old)
*Depression was more important than bone health*	
8	‘[Depression is more important because bone health] hasn't raised any health concerns’ (Female, 84 years old)
9	‘…it's [bone health] somewhere after that [depression], because I have never, ever really given it a thought [as a priority]’ (Male, 77 years old)
15	‘[Depression is priority] because I think it affect's one's happiness, it affects your enjoying the moment of life…this is life, it's not a rehearsal’ (Female, 63 years old)
16	‘[Depression is a priority] just because it affects everything…When I'm well from that [depression], then I exercise and I garden and I do stuff and I'm busy…Everything works better…The bone health, this will mend. I can deal with it. I will take steps to improve it, but it doesn't feel like a catastrophe. It feels much more manageable actually…I've done minor research around bone health and I know that it's never completely too late to start improving it…I figure that bone health is really manageable in a way that depression sometimes doesn't feel manageable’ (Female, 56 years old)
17	‘bone health would take a much secondary seat to making sure that I wasn't depressed…[bone health is] basically negligible…because I don't think my future will be encumbered by bone health problems…[Depression] has such a large impact on my life. It's the difference between functioning and not functioning. It affects everything’ (Female, 58 years old)
19	‘I have to put the depression first…it will always be the most important…[depression is the most important condition because] I wouldn't be able to care for myself. If I got really bad, I wouldn't care about doing anything for my bone health. So, I know that I have to keep that to be able to do anything else’ (Female, 61 years old)
25	‘Right now, [the depression] is more important than the bone health, because it's always a potential. And the bone health is something that…just came up’ (Male, 57 years old)
*Chronic nonfracture pain was all‐consuming*	
2	‘[the arthritis] tends to be a little bit volatile…I have it in different areas of the body’ (Female, 69 years old)
4	‘I've had rheumatoid [arthritis] since I was a child’ (Female, 52 years old)
6	Reports having had psoriatic arthritis since 2007: ‘It would take me until 4:00 in the afternoon just to make a pot of tea. I was pretty well crippled with that’ (Female, 65 years old)
10	‘The last 20 years, I've had this sciatica thing…you do something stupid and it cuts in and lays you low for two or three months…sometimes, I would wake up in the morning and I'd think, oh my lord, what's that pain there…sometimes it stays all day’ (Male, 75 years old)
11	‘My hands, I can't sometimes do things with my hands, like I can't open jars, that kind of thing. I don't have the strength anymore [due to arthritis]…it hurts every time I stand up. It [bursitis pain] lets me know it's there’ (Female, 74 years old)
12	‘my back is a mess [due to stenosis, spinal fusion, arthritis]…I have pain almost all the time, day and night…as soon as I get up to walk, I have pain’ (Female, 84 years old)
14	‘[The arthritis] seems to be in my hands. I have pain in my shoulders at times and in different places…and the back, I have back aches’ (Female, 77 years old)
19	‘I have bursitis in my right hip…[and] arthritis in my knees and my ankles…they do hurt…on a daily basis…I am getting worried about arthritis because things just hurt so much…it [pain] certainly spoils the enjoyment of, just, even walking…It's getting so it's constant’ (Female, 61 years old)
21	‘I've had arthritis my whole life since I'm 13…I look at it [pain] as a disability. There are things that I just cannot do, that I need help for…I have trouble lifting my left leg…my husband…he has to help me into the car. Even getting into bed I have to lift my leg…either he lifts my leg or I have to lift it myself with my hands, getting out of bed, putting on my pants’ (Female, 71 years old)
22	‘I get these most horrible cramps after eating, and I get explosive diarrhea…it flares, it comes and goes…sometimes I have really bad days, and sometimes it doesn't bother me for weeks or months, and then bang, it's right back in my face again’ (Male, 62 years old)
23	‘I'm not trying to be a wuss, but I feel that I have had my share of pain in this life…every one of these joints have flared up at one time or another…there is a lot of pain…the migraines are excruciating…It [pain] inhibits my productivity. I'm having trouble…writing…the pen will fall right out of my hand. I'm having trouble holding on to utensils…while I'm eating, suddenly a fork slips and lands on the floor…I have to get my bread sliced now. I can't slice it. That's a minor detail but it's exemplary of a lot of things…I can put the laundry in, and very carefully, put it in the dryer…but I can't carry the basket back up’ (Female, 80 years old)
24	pain from genital herpes makes her ‘miserable’ (Female, 59 years old)
25	‘There are days when I'm struggling to get up and walk up stairs’ (Male, 57 years old)
*Chronic nonfracture pain was more important than bone health*	
2	‘…ongoing mobility, that is the thing that I am concerned about at the moment’ (Female, 69 years old)
3	‘The pain from the fracture isn't as bad as the pain from my back’ (Female, 78 years old)
4	‘My rheumatoid arthritis [is my first priority]. It's impacting me probably the most right now. Because when I get up in the morning, I'm stiff and sore for the first hour or so. So, going somewhere early in the morning, I have to get myself up earlier…With the arthritis, if my knee is swollen, it affects me immediately. You can see it's swollen and you can't move it and I can't walk…whereas, the bone, I'm not seeing it. I don't see the deterioration so I don't really think about it’ (Female, 52 years old)
6	‘I'm starting to feel it [pain] in my knee more lately…I don't have time for nothing else, I need a break’ (Female, 65 years old)
12	‘My wrist [fracture] is fine…It's the rest of my body that I'm not sure…my wrist [fracture] is nothing compared to [my back pain]’ (Female, 84 years old)
21	‘The stiffness of arthritis is one of the biggest, biggest problems. When you get up in the morning, it takes you five minutes to just stand up and walk from the bed to the bathroom because you're just so, so stiff…we were walkers…and I'm just not able to do that. So for me, [the rheumatoid arthritis pain] is a priority because it has impacted on our quality of life that way’ (Female, 71 years old)
22	‘I'm not going to worry about diet and bone health right now…I'm trying…to get the whole gastrointestinal system, right down to the bowel, under control before I start experimenting with diets for other aspects of my health’ (Male, 62 years old)
23	‘I would say pain is my biggest enemy because I have had so much of it for so many years…I think the osteoporosis is being looked after…. but the pain of the arthritis is hard to get away from’ (Female, 80 years old)

#### 3.1.1 Depression was all‐consuming

Many participants with depression reported experiencing depression throughout their lifetime and being treated with antidepressants. For example, participant #16 said, ‘I've been diagnosed [with depression] kind of on and off since young childhood’. Participant #18 described her diagnosis of diabetes as a teenager as linked with her depression onset. She reported that the reaction of friends, family and coworkers to her putting ‘holes in [her] finger’ and taking injections every day contributed to her depression (ID18).

Participants' depression was described as all‐consuming. It was described as ‘pouring rain’ (ID23), ‘a cloud’ and ‘always there’ (ID25). One participant said, ‘the whole reason that I was referred to [psychiatrist] is because I just wanted to die…with all these conditions…it's like, oh, why me? Is it never going to stop?’ (ID5). Another participant told us about his intention to commit suicide years previously. He said, ‘I had decided that I'm just going to come home and kill myself. I mean, this came out of depression’ (ID9). One participant said she felt she had become more isolated because of her depression and had had to give up activities. She said, ‘it's kind of like with all the health problems and then you can't get out of the house and you can't get out with your friends…You can't walk as far…it's like you're losing bits and pieces’ (ID4). Participant #16 talked about how the all‐consuming nature of depression was a ‘gradual process’. She said, ‘my own particular depression manifests with fatigue and inertia and kind of deep self‐contempt. You slip into this certain knowledge that you are an absolute failure of every sort. Terrible mother, terrible scholar, useless scholar, can't even keep the house clean’.

One 58‐year‐old woman described the hopelessness that accompanied depression. She said, ‘[the depression] has tainted everything. It's ruined it. I was a very successful career woman with a very big life, lots of interests, lots of friends…and now I'm just [an] aging woman with a middle wage job, hides in her apartment’ (ID17). She described how the depression was ‘there’ even on good days, ‘you know that you're a hair's breadth away from not feeling good, always. You're always a little bit conscious of managing your surroundings and trying to stay cheerful’. She continued, ‘It's hard to describe depression to people who've never experienced it…It's a lack of hope and so the world becomes insignificant and you don't believe that anything that you do can have any impact on making your life better…you just checked out’ (ID17). One 80‐year‐old woman said that she didn't ‘seem to be capable of feeling joy’ and that ‘a good day would be that [she was] not reduced to tears’ (ID23).

#### 3.1.2 Depression was more important than bone health

Participants talked about their depression as being more important than their bone health. In fact, many participants reported they could only take care of other health conditions, including bone health, when their depression was under control. Participant #25 said his depression was currently his most important health concern because it was ‘always a potential …and the bone health [was] something that…just came up’. Participant #16 talked about her depression and anxiety as being her number one health priority because it affected everything else: ‘when I'm well…then I exercise and I garden and I do stuff and I'm busy…everything works better’. She continued, ‘bone health is really manageable in a way that depression sometimes doesn't feel manageable’ (ID16). Another participant described her depression as her priority because it affected her ‘enjoying the moments of life…this is life, it's not a rehearsal’ (ID15). Another participant told us that ‘bone health would take a much secondary seat to making sure [she] wasn't depressed’ (ID17) and participant #19 said, ‘if I got really bad, I wouldn't care about doing anything for my bone health’.

#### 3.1.3 Chronic nonfracture pain was all‐consuming

Participants described having chronic pain for 15 (ID6) to approximately 60 years (ID21; ID23). Existing chronic pain dominated the conversation when we asked participants about their fracture pain. One participant described the pain from her hip bursitis and arthritis as occurring on ‘a daily basis’ (ID19). She also talked about her pain as spoiling ‘the enjoyment of, just, even walking’. Participant #25 with osteoarthritis of the knees said, ‘there are days when I'm struggling to get up and walk’. One 65‐year‐old woman said ‘it would take me until 4:00 in the afternoon just to make a pot of tea. I was pretty well crippled with that [psoriatic arthritis]’ (ID6). One participant with osteoarthritis who had also developed bursitis at the time of the follow‐up interview said, ‘I can't sometimes do things with my hands, like I can't open certain jars…I don't have the strength anymore…it hurts every time I stand up’ (ID11). One 84‐year‐old woman with spinal stenosis said ‘my back is a mess…I haven't been able to do anything more than be in pain for the last eight months…I have pain almost all the time, day and night…as soon as I get up to walk, I have pain’ (ID12). Another participant reporting osteoarthritis and bursitis similarly said, ‘I am getting worried about arthritis because things just hurt so much’ (ID19). Participant #23 said, ‘I have had my share of pain in this life…the migraines are excruciating’. She also reported having degenerative disc disorder and osteoarthritis and said, ‘every one of these joints have flared up at one time or another’. At the time of the interview, she reported pain in her hands, ‘I'm having trouble…writing…the pen will fall right out of my hand. I'm having trouble holding onto utensils…while I'm eating, suddenly a fork flips and lands on the floor’.

#### 3.1.4 Chronic nonfracture pain was more important than bone health

Some participants described their existing chronic pain as the most concerning condition at the time of the interview. Participant #6 said, ‘I'm starting to feel it [pain] in my knee more lately…I don't have time for nothing else’. Participant #23 told us, ‘I would say pain is my biggest enemy because I have had so much of it for so many years’. Chronic nonfracture pain was considered to be a priority over bone health because it had a more immediate effect on quality of life. For example, participant #4 described her rheumatoid arthritis as the condition currently having the most impact. She said, ‘my rheumatoid arthritis. It's impacting me probably the most right now. Because when I get up in the morning, I'm stiff and sore for the first hour or two. So, going somewhere early in the morning, I have to get myself up earlier’. Later, she said, ‘with the arthritis, if my knee is swollen, it affects me immediately. You can see it's swollen and you can't move it and I can't walk, whereas the bone, I'm not seeing it. I don't see the deterioration so I don't really think about it’. Another participant said that she was taking bone active medication and knew ‘what the situation [was regarding her bone health]’ but that the pain from her rheumatoid arthritis had curtailed her walking and so it was prioritized over bone health (ID21).

Two participants minimized their fracture pain in the context of chronic pain from other conditions. One individual reporting chronic back pain told us, ‘the pain from the fracture isn't as bad as the pain from my back’ (ID3). Another participant with spinal stenosis told us, ‘my wrist [fracture pain] is fine…it's the rest of my body that I'm not sure…my wrist [fracture] is nothing compared to [my spinal stenosis]’ (ID12).

### 3.2 The fracture exacerbated reported experiences of existing depression and chronic pain

Most participants described their recent fracture as exacerbating their existing depression and/or chronic pain (see Table 5). Participants talked about how the recent fracture affected their depression. Participant #4 reported that the fracture worsened her depression because of the ‘lack of mobility, lack of being able to get out. I have to keep my mind busy’. One participant (ID9) said that having to wear a cast made his depression worse, ‘I get so tired of this [cast]…I get very angry with it’. One 63‐year‐old woman said that her recent wrist fracture had affected her depression. She said, ‘it was kind of depressing over Christmas…I was feeling dragged down because I felt sort of useless, and it [wrist] was irritating. I didn't have any freedom of movement’ (ID15). Similarly, one 58‐year‐old woman said that she had been ‘more blue than usual’ after fracturing her wrist (ID17). Participant #18 told us that her recent fracture of both wrists had affected her depression because she was now terrified of falling: ‘I find that I have no drive. I'm always sad. I can't do this and I never could do “can't”. I've always … at least attempted [things]. Now I can't do that or I've been told, “You can't do that, I won't let you do that, don't do that”. It's always negative…it weighs you down’ (ID18).

**Table 5:** The fracture exacerbated reported experiences of existing depression and chronic pain

**ID**	**Quote**	
*The fracture exacerbated reported experiences of existing depression*		
4	‘Yeah, [the fracture has] sort of aggravated it [depression]. It's almost like my depression, what do you say, tends to be affected by…lack of mobility, lack of being able to get out. I have to keep my mind busy’ (Female, 52 years old)	
8	‘[mental health affected by hip fracture because] I'm not walking much now’ (Female, 84 years old)	
9	‘I get so tired of having this [cast]…I get very angry with it’ (Male, 77 years old)	
15	‘I think [the fracture] has put me back; it's made me feel … it was kind of depressing over Christmas, and difficult. It was painful, and I couldn't do things I wanted to do…I was feeling dragged down because I felt sort of useless, and it was irritating. I didn't have any freedom of movement…it [fracture] did pull me down somewhat’ (Female, 63 years old)	
17	‘I've been more blue than usual. I do have to concede I have been more blue than usual the last couple of months and that [fracture] may have had something to do with it’ (Female, 58 years old)	
18	‘I find I have no drive. I'm always sad. I can't do this [because of the fracture] and I never could do “can't”. I've always, well at least I attempted it. Now I can't do that or I've been told you can't do that, I won't let you do that, don't do that. It's always negative. So when you get so much negative on you it weighs you down’. (Female, 45 years old)	
22	‘The…thing that has made me depressed during the experience of the fracture is the chronic pain…The pain, when it was at its worst, yeah, I felt pretty down about that’ (Male, 62 years old)	
23	‘The pain [of the fracture] makes [the depression] worse. Anything that confines me to the house makes it worse…[The fracture has] reduced me to tears almost every day. I worry because it's just one more thing for my children to worry about. I'm very concerned about their needs and not wanting to be a burden on them’. (Female, 80 years old)	
*The fracture exacerbated reported experiences of existing chronic pain*		
3	‘I'm doing these exercises [for the fracture] and sometimes I wake up in the night and I just hurt like the dickens…I think they [exercises for the fracture] might aggravate my back problem. Some mornings, I can hardly get up I'm so achy’ (Female, 78 years old)	
4	‘I can't use crutches [for the fracture] like most people…other people have strength in their arms…Because of the arthritis [pain],…I don't have the strength to use other parts of my body to help me in manoeuvering and getting around’ (Female, 52 years old)	
10	‘[The specialist] spent a while looking at it and well no, we can't see it, it's actually become dislodged. But that was a time when I went through an incredible amount of pain on that leg. And then, as these things do, it gradually went away. But it has never … it's one of these things, I'm going to have it for life, I guess…he [surgeon] actually said to me, “Well we've put this…bar or rod in there, and because you were compromised before…this goes into where the problem was [location of pain from sciatica]"…So, it's always going to be a sort of a grumbling thing down there’ (Male, 75 years old)	
11	‘ I have [osteoarthritis] in my hands and I have it in some of my joints and after the break apparently it's a bit more vulnerable’. (Female, 74 years old)	
12	‘I'm walking worse right now because I've had so much pain [fall resulting in fracture has worsened her spinal stenosis]. I'm sitting this way because if I sit like this, I don't have pain, but as soon as I get up, I have pain’. (Female, 84 years old)	
19	‘The bursitis has [worsened], because it really stopped when I started swimming regularly…I noticed that my bursitis was much, much better when I was swimming a couple of times a week. So, now it's back [bursitis is back because she is unable to swim due to the fracture]’ (Female, 61 years old)	
21	‘I've had a bit of a struggle…. because my left leg…they [rehabilitation specialists] feel that when I fell, I had a fairly severe injury [that has worsened her mobility beyond that caused by rheumatoid arthritis]’. (Female, 71 years old)	
23	‘Of course, every pain [such as the fracture pain] aggravates my osteoarthritis because that's pretty well generalized…I guess the stress of all of this put me into migraine alley again for a while’ (Female, 80 years old)	

Participant #23 discussed how her fractured pelvis added to her depression in two ways. First, the fracture increased her sense of isolation, which contributed to her depression. She said, ‘anything that confines me to the house makes it [depression] worse’. Second, she worried about being a burden to her children. She said, the fracture ‘reduced me to tears almost every day. I worry because it's just one more thing for my children to worry about…I'm very concerned about…not wanting to be a burden on them’.

Reports from three participants did not fit with our findings as they reported that their recent fracture had not affected their depression (ID16; ID19; ID25). One woman said that she ‘was entitled to be miserable’ after her wrist fracture and did not feel the fracture had triggered a period of depression (ID16). One 61‐year‐old woman said, ‘I don't think it [wrist fracture] did [affect my depression], and that's what I say about it [depression] being unpredictable’ (ID19). Participant #25 reported he was surprised that his fracture had not made his depression worse. It is of interest that the three patients who did not perceive their fracture to exacerbate their depression had sustained wrist fractures, which did not appear to result in challenges to their mobility.

Participants also talked about how the recent fracture had affected their existing chronic pain. One participant told us that the exercises prescribed for her fracture worsened her back pain. She said, ‘I'm doing these exercises and sometimes I wake up in the night and I just hurt like the dickens’ (ID3). One 75‐year‐old man with a broken femur suggested that the surgery for his fracture had aggravated his sciatic pain (ID10). He said the surgeon had said ‘well, we've put this…bar or rod in there, and because you were compromised before…this goes into where the problem [pain from sciatica] was’. One 74‐year‐old woman who had broken her shoulder talked about how the break aggravated the osteoarthritis pain in her hand (ID11). Another participant perceived that her wrist fracture had made her spinal stenosis worse. It appeared that the fall, not the wrist fracture, may have affected her gait, ‘I'm walking worse right now because I've had so much pain [related to the spinal stenosis]’ (ID12). Fear of falling and breaking another bone prevented her from doing activities to keep her back mobile and minimize her spinal stenosis pain. Similarly, participant #19 talked about how, before her fracture, swimming was the only strategy that alleviated her bursitis pain. She had been unable to swim since the fracture and her bursitis pain had increased. One participant suggested that using crutches after her fracture was difficult and heightened the pain from her rheumatoid arthritis. She said, ‘I can't use crutches [for the fracture] like most people would…other people have strength in their arms’ (ID4). Participant #21, who also had rheumatoid arthritis, talked about how her hip fracture worsened her mobility beyond that caused by her rheumatoid arthritis. She had trouble lifting her leg and her husband had to help her into the car. During the second interview, participant #23 told us she had sustained another fracture, which was aggravating her osteoarthritis pain. The stress of this new fracture had also triggered more frequent severe migraines, a condition she reported during the intake questions.

## 4 DISCUSSION

In our study of patients with a fragility fracture and other health conditions, most patients reported depression or chronic nonfracture pain and several lived with both depression and chronic pain. These two conditions were considered to be all‐consuming and were prioritized over bone health. Patients also indicated that the fragility fracture had worsened their depression and chronic pain. Our results overall suggest that depression and chronic pain are often present in the setting of fragility fracture, that they have important consequences, and that they may be under‐recognized and undertreated, especially chronic pain.

Our qualitative study was not designed to assess prevalence, and may have inadvertently selected for individuals at risk for mental health conditions (to be eligible, participants had to report one or more comorbidities in addition to compromised bone health). Nevertheless, the high proportion of patients in our study reporting depression is worrisome because depression has been associated with poor adherence to osteoporosis medication^32^ and increased falls.^59^ The experience of sustaining a fracture has been documented to precede depression,^5^, ^6^ so it is not surprising that *existing* depression may be exacerbated by the fracture. However, our results suggest that the potential implications of these relationships may be overlooked in clinical settings and may have negative consequences for bone health. The relationship between depression and bone health is clearly complex and the themes identified in our research need additional attention in future studies. For example, future studies should explore more explicitly individuals' perceptions of the effort and energy devoted to bone health in the face of depression and chronic nonfracture pain, as well as the emotional implications of dealing with all three.

Older adults with chronic pain often alter or reduce their social and physical activities in some way to avoid pain.^60^ This is a rational response to prevent further pain but it might paradoxically result in deconditioning and social isolation.^60^ A reduction in physical activity has implications for postfracture recovery, which often involves physiotherapy exercises. As demonstrated by our findings, social isolation may also aggravate existing depression. Various safe medication treatments exist for chronic pain in older adults but less than one‐half of patients with chronic pain in our study reported taking medication for their pain. This is perhaps not surprising, given that older adults are reluctant to take pain medication for conditions such as osteoarthritis, and when they do, they take the pain medication at a lower dose or frequency than prescribed.^61^ We did not ask participants if they had declined offers of pain medication or if they had not been offered medication for their chronic pain so cannot comment on why less than one‐half reported taking medication for their pain. Future studies are needed to examine in detail changes in pain and pain medication. Chronic pain is not easily treated and treatments may become less effective over time, such as in the case of opioid‐induced hyperalgesia.^62^

Our findings have important implications for clinical practice. Treatment for fractures involves a combination of nonoperative and surgical management, pain management and rehabilitation. A fracture event is typically not considered an opportunity to investigate mental health issues or other sources of chronic pain. Yet the perspectives of our patients encourage health care providers to ask initial questions about depression and pain, and taking other steps as appropriate, including referral to mental health care. This might add time and complexity, but we anticipate that it would allow exploration of issues faced by patients who have a fracture, and also promote more successful recovery from the fracture and ongoing bone health management. Further, patients who have had a fragility fracture may not recognize that fractures, especially those that result in challenges to mobility, might worsen their mood and pain. From a holistic perspective, we suggest that health care providers help patients appreciate that bone health treatment prevents subsequent fractures, which, in turn, may prevent the worsening of unpleasant subjective states that matter to patients.

The main limitation of our study is that the downstream effects of the fragility fracture on depression and pain remain unclear as we did not follow patients for longer than 1 year. We did not collect information on adherence to bone health recommendations so cannot comment on whether pain and depression affected the uptake of these recommendations. We also did not collect information on body mass index so cannot comment on the link between it and bone health. Further, we did not ask whether patients were depressed or had chronic pain before previous fragility fractures. A diagnosis of depression and chronic pain was based on self‐report. We did not measure depression (or depressive symptoms) or pain and we did not determine the course of symptoms so cannot comment on the severity of these conditions. However, the reported prescriptions for medication in patients with depression and chronic pain suggest these conditions were diagnosed by a health care provider and the quotes we have provided demonstrate the apparent impact of these conditions. We conclude that these quotations support the findings and promote that the study maintains a patient perspective. Another strength of our study was the use of a phenomenological approach, which was well‐suited to our objectives to collect data on patient's experiences. Finally, two independent qualitative researchers coded and analysed the data and we drew on the expertise of the research team, some of whom have previously collaborated in investigating the relationship between depression and pain (J. E. M. S., M. G., S. T., G. H.).^14^, ^61^, ^63^, ^64^

## 5 CONCLUSION

Pre‐existing depression and chronic pain appear to be common in individuals with a fragility fracture. Patients expressed that experiences with depression and pain were prioritized over bone health and in fact, worsened as a result of the fracture. Health care providers who treat fragility fractures might ask their patients about existing depression and chronic pain and take appropriate steps to address patients' more general emotional and physical state. Interventions might emphasize that better bone health can promote not only physical but also psychological well‐being.

## AUTHOR CONTRIBUTIONS

Joanna E. M. Sale made substantial contributions to conception and design and analysis and interpretation of the data, drafted and revised the manuscript critically for important intellectual content, approved the version of the manuscript submitted and agreed to be accountable for all aspects of the work. Monique Gignac made substantial contributions to conception and design and analysis and interpretation of the data, revised the manuscript critically for important intellectual content, approved the version of the manuscript submitted and agreed to be accountable for all aspects of the work. Lucy Frankel made substantial contributions to the acquisition of data and analysis and interpretation of the data, revised the manuscript critically for important intellectual content, approved the version of the manuscript submitted and agreed to be accountable for all aspects of the work. Stephen Thielke made substantial contributions to the analysis and interpretation of the data, revised the manuscript critically for important intellectual content, approved the version of the manuscript submitted and agreed to be accountable for all aspects of the work. Earl Bogoch made substantial contributions to conception and design and analysis and interpretation of the data, revised the manuscript critically for important intellectual content, approved the version of the manuscript submitted and agreed to be accountable for all aspects of the work. Victoria Elliot‐Gibson made substantial contributions to conception and design and analysis and interpretation of the data, revised the manuscript critically for important intellectual content, approved the version of the manuscript submitted and agreed to be accountable for all aspects of the work. Gillian Hawker made substantial contributions to conception and design and analysis and interpretation of the data, revised the manuscript critically for important intellectual content, approved the version of the manuscript submitted and agreed to be accountable for all aspects of the work. Larry Funnel made substantial contributions to conception and design and analysis and interpretation of the data, revised the manuscript critically for important intellectual content, approved the version of the manuscript submitted and agreed to be accountable for all aspects of the work.

## CONFLICT OF INTERESTS

The authors declare that there are no conflict of interests.

## Data Availability

Data available on request from the authors. The data that support the findings of this study are available from the corresponding author upon reasonable request.

## References

[1] Wu Q , Liu B , Tonmoy S . Depression and risk of fractures and bone loss: an updated meta‐analysis of prospective studies. Osteoporos Int. 2018;29(6):1303‐1312. 29532130 10.1007/s00198-018-4420-1

[2] Huang AR , Mallet L , Rochefort CM , Eguale T , Buckeridge DL , Tamblyn R . Medication‐related falls in the elderly: causative factors and preventive strategies. Drugs Aging. 2012;29(5):359‐376. 22550966 10.2165/11599460-000000000-00000

[3] American Geriatrics Society Beers Criteria® Update Expert Panel . American Geriatrics Society 2019 updated AGS Beers Criteria for potentially inappropriate medication use in older adults. J Am Geriatr Soc. 2019;67(4):647‐694. 30693946 10.1111/jgs.15767

[4] Mazziotti G , Canalis E , Giustina A . Drug‐induced osteoporosis: mechanisms and clinical implications. Am J Med. 2010;123:877‐884. 20920685 10.1016/j.amjmed.2010.02.028

[5] Chang CY , Chen WL , Liou YF . Increased risk of major depression in the three years following a femoral neck fracture—a national population‐based follow‐up study. PLoS One. 2014;9(3):e89867. 24626193 10.1371/journal.pone.0089867 PMC3953077

[6] Cristancho P , Lenze EJ , Avidan MS , Rawson KS . Trajectories of depressive symptoms after hip fracture. Psychol Med. 2016;46(7):1413‐1425. 27032698 10.1017/S0033291715002974 PMC4871603

[7] Nguyen KD , Bagheri B , Bagheri H . Drug‐induced bone loss: a major safety concern in Europe. Expert Opin Drug Saf. 2018;17(10):1005‐1014. 30222369 10.1080/14740338.2018.1524868

[8] Hegeman J , van den Bemt BJ , Duysens J , van Limbeek J . NSAIDS and the risk of accidental falls in the elderly: a systematic review. Drug Saf. 2009;32(6):489‐498. 19459716 10.2165/00002018-200932060-00005

[9] Hallberg I , Ek A‐C , Toss G , Bachrach‐Lindström M . A striving for independence: a qualitative study of women living with vertebral fracture. BMC Nurs. 2010;9:7‐16. 20398360 10.1186/1472-6955-9-7 PMC2873268

[10] Suzuki N , Ogikubo O , Hansson T . The course of the acute vertebral body fragility fracture: its effect on pain, disability and quality of life during 12 months. Eur Spine J. 2008;17:1380‐1390. 18751742 10.1007/s00586-008-0753-3 PMC2556463

[11] Ettinger B , Black DM , Nevitt MC , et al. Contribution of vertebral deformities to chronic back pain and disability. the study of Osteoporotic Fractures Research Group. J Bone Miner Res. 1992;7(4):449‐456. 1535172 10.1002/jbmr.5650070413

[12] Nevitt MC , Ettinger B , Black DM , et al. The association of radiographically detected vertebral fractures with back pain and function: a prospective study. Ann Intern Med. 1998;128(10):793‐800. 9599190 10.7326/0003-4819-128-10-199805150-00001

[13] Sale JEM , Frankel L , Thielke S , Funnell L . Pain and fracture‐related limitations persist 6 months after a fragility fracture. Rheumatol Int. 2017;37:1317‐1322. 28634634 10.1007/s00296-017-3761-y

[14] Gheorghita A , Webster F , Thielke S , Sale J . Long‐term experiences of pain after a fragility fracture. Osteoporos Int. 2018;29:1093‐1104. 29455247 10.1007/s00198-018-4399-7

[15] Lindau T , Adlecreutz C , Aspenberg P . Cartilage injuries in distal radial fractures. Acta Orthop Scand. 2003;74(3):327‐331. 12899554 10.1080/00016470310014265

[16] McKinley TO , Borrelli J Jr , D'Lima DD , Furman BD , Giannoudis PV . Basic science of intra‐articular fractures and posttraumatic osteoarthritis. J Orthop Trauma. 2010;24(9):567‐570. 20736796 10.1097/BOT.0b013e3181ed298d PMC3662545

[17] Majumdar SR , Rowe BH , Folk D , et al. A controlled trial to increase detection and treatment of osteoporosis in older patients with a wrist fracture. Ann Intern Med. 2004;141(5):366‐373. 15353428 10.7326/0003-4819-141-5-200409070-00011

[18] Rucker D , Rowe BH , Johnson JA , et al. Educational intervention to reduce falls and fear of falling in patients after fragility fracture: results of a controlled pilot study. Prev Med. 2006;42(4):316‐319. 16488469 10.1016/j.ypmed.2006.01.008

[19] Kelly‐Pettersson P , Samuelsson B , Unbeck M , et al. The influence of depression on patient‐reported outcomes for hip‐fracture patients 1 year after surgery: a prospective cohort study. Aging Clin Exp Res. 2020;32:247‐255. 31028625 10.1007/s40520-019-01207-5 PMC7033144

[20] WHO Organization Assessment of fracture risk and application to screening for postmenopausal osteoporosis (WHO Technical Report Series). World Health Organization. 1994. 7941614

[21] Mackey DC , Lui L‐Y , Cawthon PM , et al. High‐trauma fractures and low bone mineral density in older women and men. JAMA. 2007;298(20):2381‐2388. 18042915 10.1001/jama.298.20.2381

[22] Langsetmo L , Goltzman D , Kovacs CS , et al. Repeat low‐trauma fractures occur frequently among men and women who have osteopenic bone mineral density. J Bone Miner Res. 2009;24(9):1515‐1522. 19338456 10.1359/jbmr.090319 PMC5101057

[23] van Geel TACM , van Helden S , Geusens PP , Winkens B , Dinant GJ . Clinical subsequent fractures cluster in time after first fractures. Ann Rheum Dis. 2009;68:99‐102. 18677009 10.1136/ard.2008.092775

[24] Mitchell PJ , Cooper C , Fujita M , et al. Quality improvement initiatives in fragility fracture care and prevention. Curr Osteoporos Rep. 2019;17:510‐520. 31734907 10.1007/s11914-019-00544-8

[25] Papaioannou A , Morin S , Cheung AM , et al. Clinical practice guidelines for the diagnosis and management of osteoporosis in Canada. Can Med Assoc J. 2010;182(17):1864‐1873. 20940232 10.1503/cmaj.100771 PMC2988535

[26] Bawa HS , Weick J , Dirschl DR . Anti‐osteoporotic therapy after fragility fracture lowers rate of subsequent fracture. J Bone Joint Surg. 2015;97:1555‐1562. 26446962 10.2106/JBJS.N.01275

[27] Bergman J , Nordstrom A , Nordstrom P . Bisphosphonate use after clinical fracture and risk of new fracture. Osteoporos Int. 2018;29:937‐945. 29397408 10.1007/s00198-017-4367-7 PMC5854733

[28] Lyles KW , Colón‐Emeric CS , Magaziner JS , et al. Zoledronic acid and clinical fractures and mortality after hip fracture. N Engl J Med. 2007;357(18):1799‐1809. 17878149 10.1056/NEJMoa074941 PMC2324066

[29] Skjødt MK , Khalid S , Ernst M , et al. Secular trends in the initiation of therapy in secondary fracture prevention in Europe: a multi‐national cohort study including data from Denmark, Catalonia, and the United Kingdom. Osteoporos Int. 2020;31:1535‐1544. 32185437 10.1007/s00198-020-05358-4 PMC7360649

[30] Balasubramanian A , Tosi LL , Lane JM , Dirschl DR , Ho PR , O'Malley CD . Declining rates of osteoporosis management following fragility fractures in the U.S., 2000 through 2009. J Bone Joint Surg. 2014;96(51‐58):e52. 24695929 10.2106/JBJS.L.01781

[31] Sale JE , Beaton D , Posen J , Elliot‐Gibson V , Bogoch E . Systematic review on interventions to improve osteoporosis investigation and treatment in fragility fracture patients. Osteoporos Int. 2011;22(7):2067‐2082. 21607808 10.1007/s00198-011-1544-y

[32] Yeam CT , Chia S , Tan HCC , Kwan YH , Fong W , Seng J . A systematic review of factors affecting medication adherence among patients with osteoporosis. Osteoporos Int. 2018;29:2623‐2637. 30417253 10.1007/s00198-018-4759-3

[33] Seaman AT , Steffen M , Doo T , Healy HS , Solimeo SL . Metasynthesis of patient attitudes toward bone densitometry. J Gen Intern Med. 2018;33:1796‐1804. 30054881 10.1007/s11606-018-4587-3 PMC6153231

[34] Sale JE , Beaton DE , Sujic R , Bogoch ER . “If it was osteoporosis, I would have really hurt myself”. Ambiguity about osteoporosis and osteoporosis care despite a screening program to educate fracture patients. J Eval Clin Pract. 2010;16(3):590‐596. 20102434 10.1111/j.1365-2753.2009.01176.x

[35] Sale JE , Gignac MA , Hawker G , et al. Decision to take osteoporosis medication in patients who have had a fracture and are ‘high’ risk for future fracture. BMC Musculoskelet Disord. 2011;12:92. 21554729 10.1186/1471-2474-12-92 PMC3103493

[36] Meadows LM , Mrkonjic LA , O'Brien MD , Tink W . The importance of communication in secondary fragility fracture treatment and prevention. Osteoporos Int. 2007;18:159‐166. 16983457 10.1007/s00198-006-0213-z

[37] Sale JE , Hawker G , Cameron C , et al. Perceived messages about bone health after a fracture are not consistent across health care providers. Rheumatol Int. 2015;35(1):97‐103. 24962740 10.1007/s00296-014-3079-y

[38] Rothmann MJ , Jakobsen PR , Jensen CM , Hermann AP , Smith AC , Clemensen J . Experiences of being diagnosed with osteoporosis: a meta‐synthesis. Arch Osteoporos. 2018;13:21. 29511831 10.1007/s11657-018-0436-6

[39] Meadows LM , Mrkonjic LA , Lagendyk LE , Petersen KM . After the fall: women's views of fractures in relation to bone health at midlife. Women Health. 2004;39(2):47‐62. 15130861 10.1300/J013v39n02_04

[40] Sale JE , Gignac MA , Frankel L , et al. Patients reject the concept of fragility fracture—a new understanding based on fracture patients' communication. Osteoporos Int. 2012;23(12):2829‐2834. 22310958 10.1007/s00198-012-1914-0

[41] Sale JEM , Gignac MA , Hawker G , et al. Patients do not have a consistent understanding of high risk for future fracture: a qualitative study of patients from a post‐fracture secondary prevention program. Osteoporos Int. 2016;27(1):65‐73. 26115943 10.1007/s00198-015-3214-y

[42] Sale JEM , Frankel L , Paiva J , et al. Having caregiving responsibilities affects management of fragility fractures and bone health. Osteoporos Int. 2020;31:1565‐1572. 32222788 10.1007/s00198-020-05385-1

[43] Giorgi A . The theory, practice, and evaluation of the phenomenological method as a qualitative research procedure. J Phenomenol Psychol. 1997;28:235‐260.

[44] Giorgi A . Concerning a serious misunderstanding of the essence of the phenomenological method in psychology. J Phenomenol Psychol. 2008;39:33‐58.

[45] Wertz FJ . Phenomenological research methods for counseling psychology. J Couns Psychol. 2005;52(2):167‐177.

[46] Moustakas C . Phenomenological research methods. Sage Publications; 1994.

[47] Polkinghorne DE . Phenomenological research methods. In: Valle RS , Halling S , eds. Existential‐Phenomenological Perspectives in Psychology. Plenum Press; 1989:41‐60.

[48] Bogoch ER , Elliot‐Gibson V , Beaton DE , et al. Effective initiation of osteoporosis diagnosis and treatment for patients with a fragility fracture in an orthopaedic environment. J Bone Joint Surg Am. 2006;88(1):25‐34. 10.2106/JBJS.E.00198 16391246

[49] Bogoch ER , Elliot‐Gibson V , Beaton D , Sale J , Josse RG . Fracture prevention in the orthopaedic environment: outcomes of a coordinator‐based fracture liaison service. J Bone Joint Surg. 2017;99:820‐831. 28509822 10.2106/JBJS.16.01042

[50] Jasper MA . Issues in phenomenology for researchers of nursing. J Adv Nurs. 1994;19:309‐314. 8188962 10.1111/j.1365-2648.1994.tb01085.x

[51] Bajcar JM , Wang L , Moineddin R , Nie JX , Tracy CS , Upshur RE . From pharmaco‐therapy to pharmaco‐prevention: trends in prescribing to older adults in Ontario, Canada, 1997‐2006. BMC Fam Pract. 2010;11:75. 20929561 10.1186/1471-2296-11-75 PMC2958963

[52] Statistics Canada . *A Portrait of Seniors in Canada* (Report no. 89‐519‐XIE). 2006:1‐301.

[53] NVivo 11 . Qualitative Solutions and Research Pty Ltd., Victoria, Australia; 2016.

[54] Kvale S . Interviews: An Introduction to Qualitative Research Interviewing. Sage Publications; 1996.

[55] Dixon‐Woods M , Shaw RL , Agarwal S , Smith JA . The problem of appraising qualitative research. Qual Saf Health Care. 2004;13:223‐225. 15175495 10.1136/qshc.2003.008714 PMC1743851

[56] Giacomini MK , Cook DJ . Users' guides to the medical literature XXIII. Qualitative research in health care B. What are the results and how do they help me care for my patients? JAMA. 2000;284(4):478‐482. 10904512 10.1001/jama.284.4.478

[57] Mason J . Qualitative Researching. 2nd. Sage Publications; 2002.

[58] Staniszewska S , Brett J , Simera I , et al. GRIPP2 reporting checklists: tools to improve reporting of patient and public involvement in research. BMJ. 2017;358:j3453. 28768629 10.1136/bmj.j3453 PMC5539518

[59] Zhou J , Xue Y . Depression, falls, and osteoporotic fractures. Osteoporos Int. 2020;31:1175. 32170397 10.1007/s00198-020-05347-7

[60] Mackichan F , Adamson J , Gooberman‐Hill R . ‘Living within your limits’: activity restriction in older people experiencing chronic pain. Age Ageing. 2013;42:702‐708. 23978405 10.1093/ageing/aft119

[61] Sale JE , Gignac M , Hawker G . How ‘bad’ does the pain have to be? A qualitative study examining adherence to pain medication in older adults with osteoarthritis. Arthritis Rheum. 2006;55(2):272‐278. 16583418 10.1002/art.21853

[62] Yi P , Pryzbylkowski P . Opioid induced hyperalgesia. Pain Med. 2015;16:S32‐S36. 26461074 10.1111/pme.12914

[63] Sale JEM , Gignac M , Hawker G . The relationship between disease symptoms, life events, coping and treatment, and depression among older adults with osteoarthritis. J Rheumatol. 2008;35:335‐342. 18203312

[64] Hawker GA , Gignac MA , Badley E , et al. A longitudinal study to explain the pain‐depression link in older adults with osteoarthritis. Arthritis Care Res. 2011;63(10):1382‐1390. 10.1002/acr.20298 20662042

